# Transcriptome profiles acquired during cell expansion and licensing validate mesenchymal stromal cell lineage genes

**DOI:** 10.1186/s13287-020-01873-7

**Published:** 2020-08-14

**Authors:** Danielle M. Wiese, Lorena R. Braid

**Affiliations:** Aurora BioSolutions Inc., Crescent Heights PO Box 21053, Medicine Hat, AB T1A 6N0 Canada

**Keywords:** Mesenchymal stromal cell, Transcriptomics, Cell characterization, Molecular profiling, Identity assays, Umbilical cord, Bone marrow, Licensing

## Abstract

**Background:**

Mesenchymal stromal cells (MSCs) are rapidly advancing as commercial therapeutics. However, there are still no adequate tools to validate the identity of MSCs and support standardization of MSC-based products. Currently accepted metrics include cell surface marker profiling and tri-lineage differentiation assays, neither of which is definitive. Transcript profiling represents a cost- and time-effective approach amenable to MSC manufacturing processes. Two independent labs recently reported non-overlapping MSC-specific transcriptomic signatures of 489 and 16 genes.

**Methods:**

Here, we interrogated our repository of transcriptome data to determine whether routine culture manipulations including cell expansion and immune activation affect expression of the reported MSC lineage genes. These data sets comprise 4 donor populations of human umbilical cord (UC) MSCs serially cultured from cryopreservation thaw through pre-senescence, and 3 donor populations each of naïve UC and bone marrow (BM) MSCs and licensed by 3 different cytokines.

**Results:**

Overall, 437 of 456 proposed signature genes assessed in these data sets were reliably expressed, representing an enduring lineage profile in 96% agreement with the previous studies. Serial passaging resulted in the downregulation of 3 signature genes, and one was silenced. Cytokine stimulation downregulated expression of 16 signature genes, and 3 were uniformly silenced in one or the other MSC type. Fifteen additional genes were unreliably detected, independent of culture manipulation.

**Conclusion:**

These results validate and refine the proposed transcriptomic tools for reliable identification of MSCs after isolation through cell expansion and after inflammatory activation. We propose a 24-gene signature to support standardized and accessible MSC characterization.

## Background

Mesenchymal stromal cells (MSC) exhibit phenotypic and functional heterogeneity related to tissue origin, donor demographics, and processing protocols. Current metrics to establish MSC identity include plastic adherence, cell surface phenotyping, and tri-lineage differentiation [1], which do not clearly distinguish MSCs from other stromal resident cells such as fibroblasts [2] or hepatic stellate cells [3]. Two research groups recently performed deep integrative analysis of publicly available transcriptomics data for multiple MSC types compared to other stem and stromal cells to generate MSC-specific signatures [4, 5]. Using combinatorial analysis of 285 samples from public data and in-house microarray and RNA-Seq data, Roson-Burgo et al. derived an MSC lineage signature of 489 genes based primarily on genes upregulated in the bone marrow (BM), adipose, and placental MSCs compared with hematopoietic stem and progenitor cells [4]. The “Rohart MSC test”, an in silico classifier based on 16 MSC signature genes, was created and validated using over 100 transcriptome studies employing 15 different quantification platforms [5]. This test reportedly distinguishes MSCs from non-MSCs with > 95% accuracy [5].

Manufacturing of MSC therapies often includes extended cell expansion or inflammatory licensing. Although these candidate genes have been validated for cell isolation protocols, their usefulness for in-process identity testing under the pressures of cell expansion and immune activation has not been formally investigated. We recently generated two transcriptome data sets by microarray analysis of 14,500 genes. The first set of 57 arrays comprises longitudinal culture of 4 umbilical cord-derived (UC) MSC populations analyzed at every passage (P) from P1 or P2 through pre-senescence [6]. The second set of 24 arrays comprises a matrix evaluation of 3 populations each of UC and BM-MSCs at rest and after priming with TNF-α, IFN-γ, or IL-1β (submitted manuscript). All MSC populations were expanded to comparable population doublings in xeno- and serum-free media, with donor sex balanced between groups. Here, to address the need for tools with validated utility across dynamic MSC biology and tissue and donor-influenced heterogeneity, we interrogated expression of the 2 proposed MSC signatures in these 2 substantial data sets that span culture medium formulation, tissue source, donor source, cell aging, and 3 canonical cell licensing conditions.

## Methods

MSCs used to generate the data sets were validated by surface marker profiling and tri-lineage differentiation and were cultured and manipulated as previously described ([6] and submitted manuscript). Briefly, cells were maintained in TheraPEAK™ MSC growth medium chemically defined (Lonza, MD, USA) and serially propagated until senescence [6] or cultivated in xeno-free human platelet lysate (hPL)-supplemented media (RoosterBio Inc., MD, USA) and then activated by a 24-h co-incubation with TNF-α (50 ng/ml), IFN-γ (50 ng/ml), or IL-1β (80 pg/ml) in protein-free media (RoosterBio Inc.; submitted manuscript).

Microarray analysis was conducted using R Bioconductor packages [7, 8]. Gene Expression Omnibus [9, 10] data sets GSE119987 and GSE129165 were previously pre-processed using simpleaffy [11], GCRMA [12] and queried for batch effects using BatchQC [13]. For transcripts mapped by multiple probes, the probe set with highest average expression intensity across all samples was used. Pairwise comparisons of P2 versus each subsequent passage within each UC-MSC population (GSE119987) and comparisons of resting versus primed UC and BM-MSCs separately (GSE129165) were performed using eBayes in limma [14, 15]. Significantly differentially expressed probe sets had a false discovery rate-adjusted *p* value (*p*) of < 0.05 with sequential filtering for > 2-fold change.

Forty-four of the 489 Roson-Burgo MSC lineage genes [4] and 5 of 16 Rohart MSC test genes [5] are not represented on the Affymetrix GeneChip Human Genome U133A 2.0 microarray and were not analyzed.

## Results

### Most MSC signature genes are stable during cell expansion

**Fig. 1:**
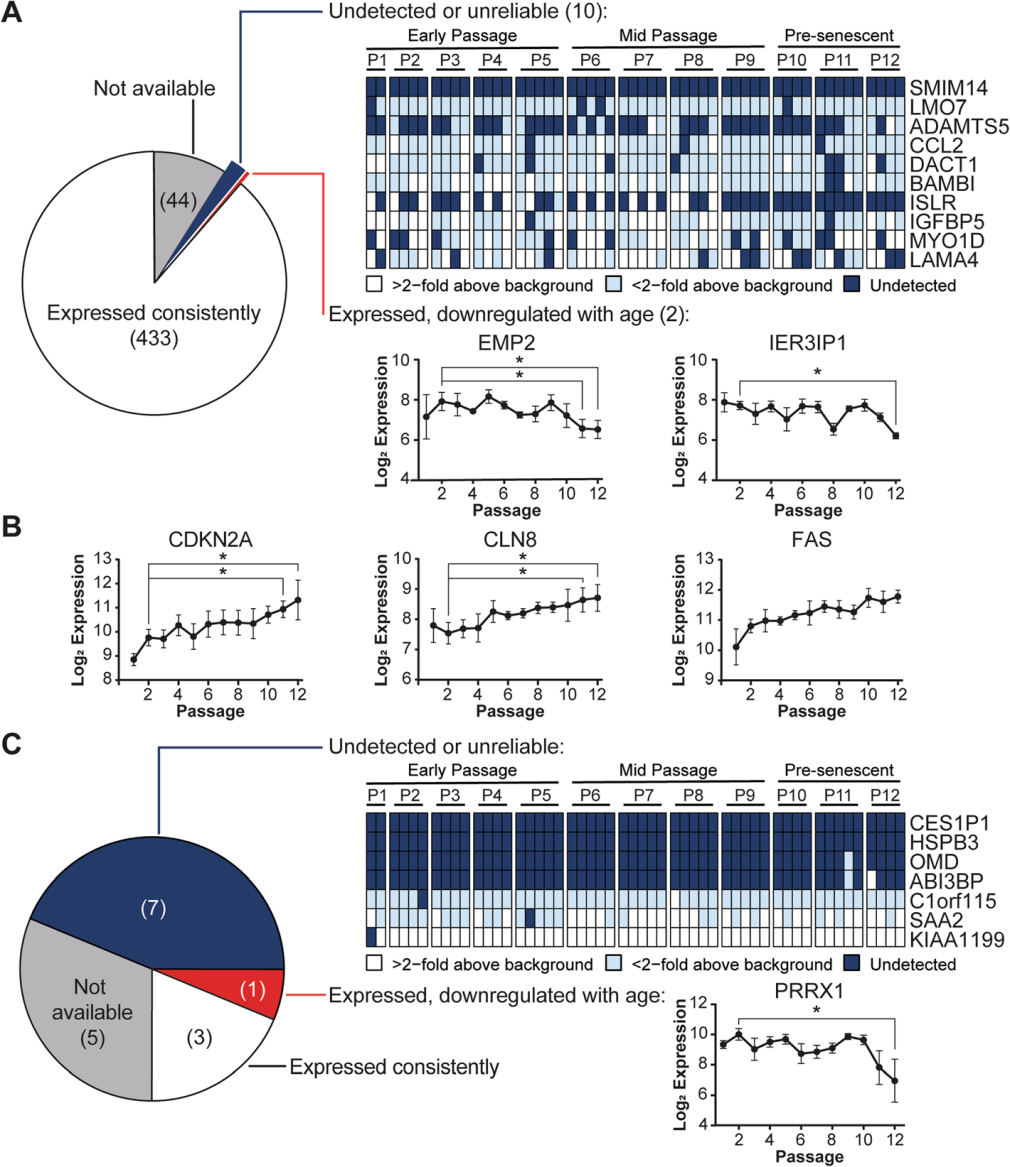
Stability of MSC lineage profiles during prolonged UC-MSC expansion. **a** Most interrogated transcripts in the Roson-Burgo MSC lineage gene set (433/445) are expressed and unchanged by extended passaging. *SMIM14* is never detected, while 9 other genes are expressed intermittently during cell propagation. *EMP2* and *IER3IP1* are downregulated with age but remain detectable. **b** Genes previously linked to senescence (*CDKN2A*) or aging (*CLN8*, *FAS*) as defined by Gene Ontology functional classification are upregulated at P10, 11, and 12 versus P2 (*p* < 0.05, > 1.5-fold change) [6]. **c** Four of the 11 tested Rohart MSC genes are reliably expressed by serially expanded UC-MSCs, although *PRRX1* expression decreases in pre-senescent cells. Four genes (*CES1P1*, *HSPB3*, *OMD*, and *ABI3BP*) are essentially undetectable. *C1orf115*, S*AA2*, and *KIAA1199* are expressed throughout cell expansion but are deemed unreliable since expression intensity occasionally falls below the detection limit. Graphs depict the mean ± SEM of biological replicates. Abbreviations: P, passage. *p < 0.05 and > 2-fold change

We recently reported that the transcriptomes of serially expanded UC-MSCs diverge from early passage counterparts in tiers of magnitude corresponding to early passage (P1–5), mid-passage (P6–9), and pre-senescence (P10-senescence) [6]. Intriguingly, UC-MSCs at any passage constitutively express 435 (98%) of 445 tested Roson-Burgo MSC signature genes [4], despite accumulating age-related markers during pre-senescence (Fig. 1a, b, Table 1, Additional file 1: Fig. S1A). These identity genes were consistently expressed during cell expansion, except for *EMP2* and *IER3IP1* which declined during pre-senescence (Fig. 1a). At P11, expression of *EMP2* decreased 2.89-fold compared to P2 (*p* = 0.046), and 3.25-fold at P12 (*p* = 0.018). *IER3IP1* expression also significantly decreased at P12 (− 2.91-fold, *p* = 0.025). However, UC-MSCs maintained moderate expression of these genes through pre-senescence. By contrast, 10 genes were unreliably expressed, with no detectable signal above microarray background in at least 1 sample (Fig. 1a). *SMIM14* was never detected, while 9 others were sporadically expressed. *ISLR* was expressed by some early to mid-passage MSC populations but was undetected after P8.

**Table 1:** Summary of MSC transcriptomic lineage marker expression by MSCs during prolonged culture or licensing

		Roson-Burgo core signature	Rohart MSC test
	Assessed	445/489 (91%)	11/16 (69%)
Passage 2–12 UC-MSCs	Reliable	435/445 (98%)	4/11 (36%)
Resting and primed UC and BM-MSCs	Reliable	436/445 (98%)	5/11 (45%)
Overlap	Reliable	433	4 (*CXCL2*, *GDF5*, *MBD2*, *PRRX1*)
	Unreliable	7 (***ADAMTS5***, ***DACT1***, *IGFBP5*, ***ISLR***, ***LAMA4***, *LMO7*, *SMIM14*)	6 (*CES1P1*, *ABI3BP*, *C1orf115*, *HSPB3*, *OMD*, *SAA2*)

Transcripts in bold are uniformly silenced under specific culture conditions

Four of the 11 available Rohart MSC test genes [5], *CXCL2*, *GDF5*, *MBD2,* and *PRRX1*, were expressed in all 57 samples during UC-MSC cell expansion (Fig. 1c, Table 1, Additional file 1: Fig. S1B). Only *PRRX1* expression dropped significantly, at P12 (− 9.38-fold versus P2, *p* = 0.040) (Fig. 1c). *C1orf115*, *SAA2*, and *KIAA1199* (*CEMIP*) were also expressed throughout cell expansion but were each undetected at least once (Fig. 1c). *CES1P1* and *HSPB3* were never detected, while *OMD* and *ABI3BP* were only measurable in some pre-senescent populations (Fig. 1c).

### Cytokine activation has little impact on expression of MSC lineage genes

**Fig. 2:**
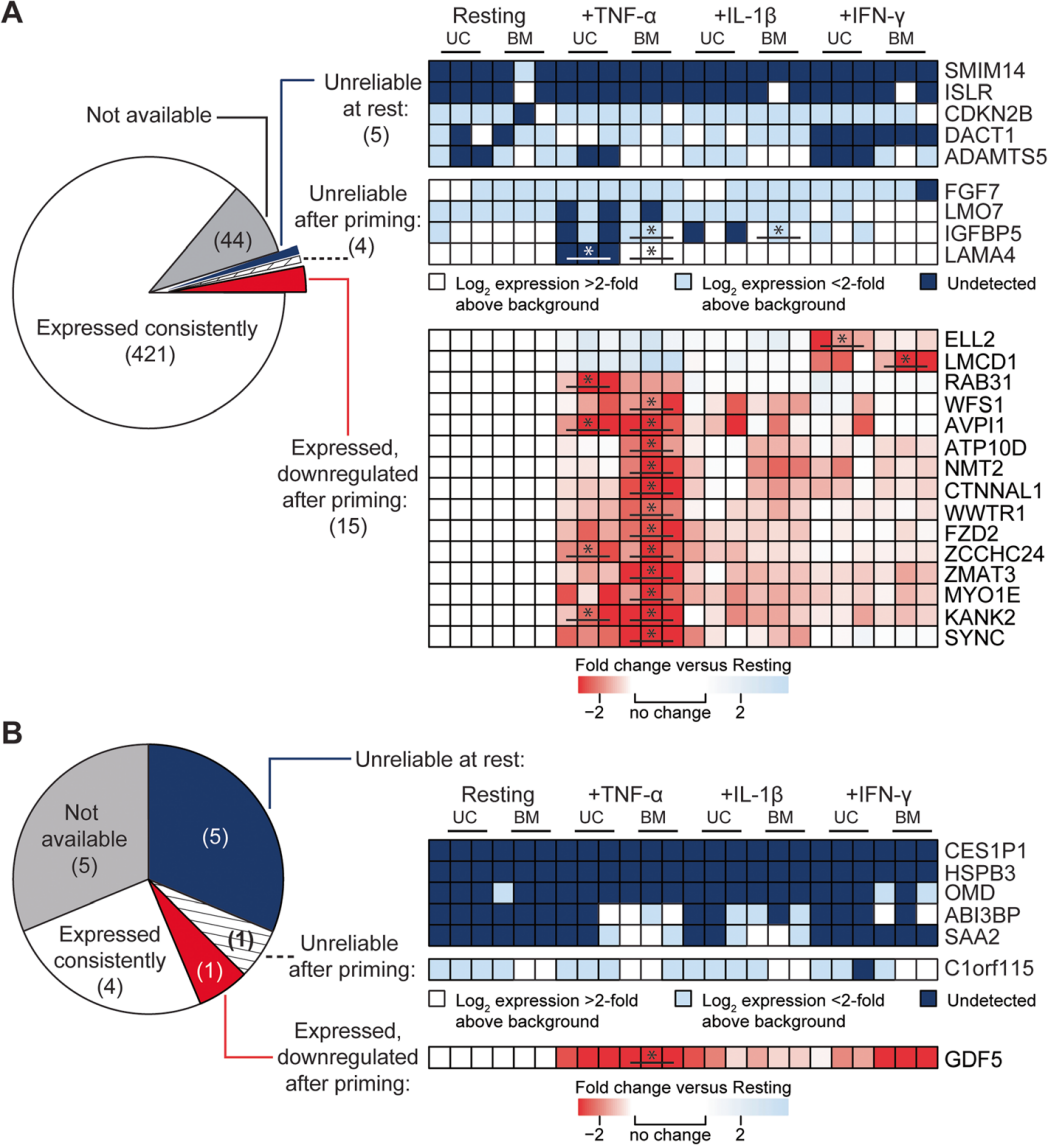
Impact of cytokine activation on MSC lineage genes in UC and BM-MSCs. **a** A small cohort of Roson-Burgo MSC lineage genes is undetected before (5/445) or after (4/445) cytokine stimulation. Of these, *DACT1*, *ADAMTS5*, and *LAMA4* are silenced in a cytokine-specific manner. Fifteen genes are significantly downregulated after conditioning by TNF-α or IFN-γ but remain detectable. **b** Four Rohart MSC test genes are consistently expressed in naïve and activated UC and BM-MSCs. In addition, *GDF5* is downregulated but still measurable in all primed MSC populations. *C1orf115* was also consistently expressed, except in one IFN-γ primed UC-MSC population. *CES1P1* and *HSPB3* were never detected, while *OMD*, *ABI3BP*, and *SAA2* are only detected in some licensed populations, predominantly BM-MSCs. *p < 0.05 and > 2-fold change

Three populations each of resting UC and BM-MSCs cultured in hPL-supplemented media (submitted manuscript) expressed 440 (99%) of 445 signature genes proposed by Roson-Burgo et al. [4] (Fig. 2a, Additional file 2: Fig. S2A). Fifteen of these 440 genes were expressed at significantly lower levels after cytokine licensing with TNF-α or IFN-γ, but were still detected (*p* < 0.05, > 2-fold change; Fig. 2a). Four genes were undetected in at least 1 population of licensed MSCs (Fig. 2a); *LAMA4* was specifically silenced in all TNF-α primed UC-MSCs (*p* = 0.024).

*ADAMTS5* and *DACT1* were detected in 4 of 6 resting MSC populations and were also influenced by inflammatory activation (Fig. 2a). *DACT1* expression was abrogated in all IFN-γ primed MSC populations, while TNF-α or IL-1β stimulated *DACT1* expression in all MSC populations. *ADAMTS5* was specifically silenced in all IFN-γ primed UC-MSCs and remained off in 2 of 3 TNF-α primed UC-MSC populations. In BM-MSCs, however, *ADAMTS5* was not affected by IFN-γ priming but increased in response to TNF-α or IL-1β activation. Notably, 7 of 9 Roson-Burgo genes unreliably detected in naïve or licensed MSCs are consistent with those found unreliable during prolonged UC-MSC expansion (Table 1).

Six of the 11 available Rohart MSC test genes [5] were expressed by all 6 resting UC and BM-MSC populations cultivated in hPL (Fig. 2b, Table 1, Additional file 2: Fig. S2B), although *GDF5* was significantly downregulated to moderate levels in TNF-α activated BM-MSCs (− 20.44-fold, *p* = 0.012). *C1orf115* became undetectable in one IFN-γ conditioned UC-MSC population (Fig. 2b). Consistent with serially expanded UC-MSCs, *CES1P1* and *HSPB3* were undetected in all MSC samples (Fig. 2b). *ABI3BP* and *SAA2* were also not detected in resting UC and-BM-MSCs but could be induced in a subset of donor populations responsive to TNF-α or IL-1β (Fig. 2b). These 5 genes, together with *OMD*, were also unreliable in serially passaged UC-MSCs (Table 1).

### A panel of 24 validated MSC lineage genes may be suited for transcript-based MSC lineage assays

**Fig. 3:**
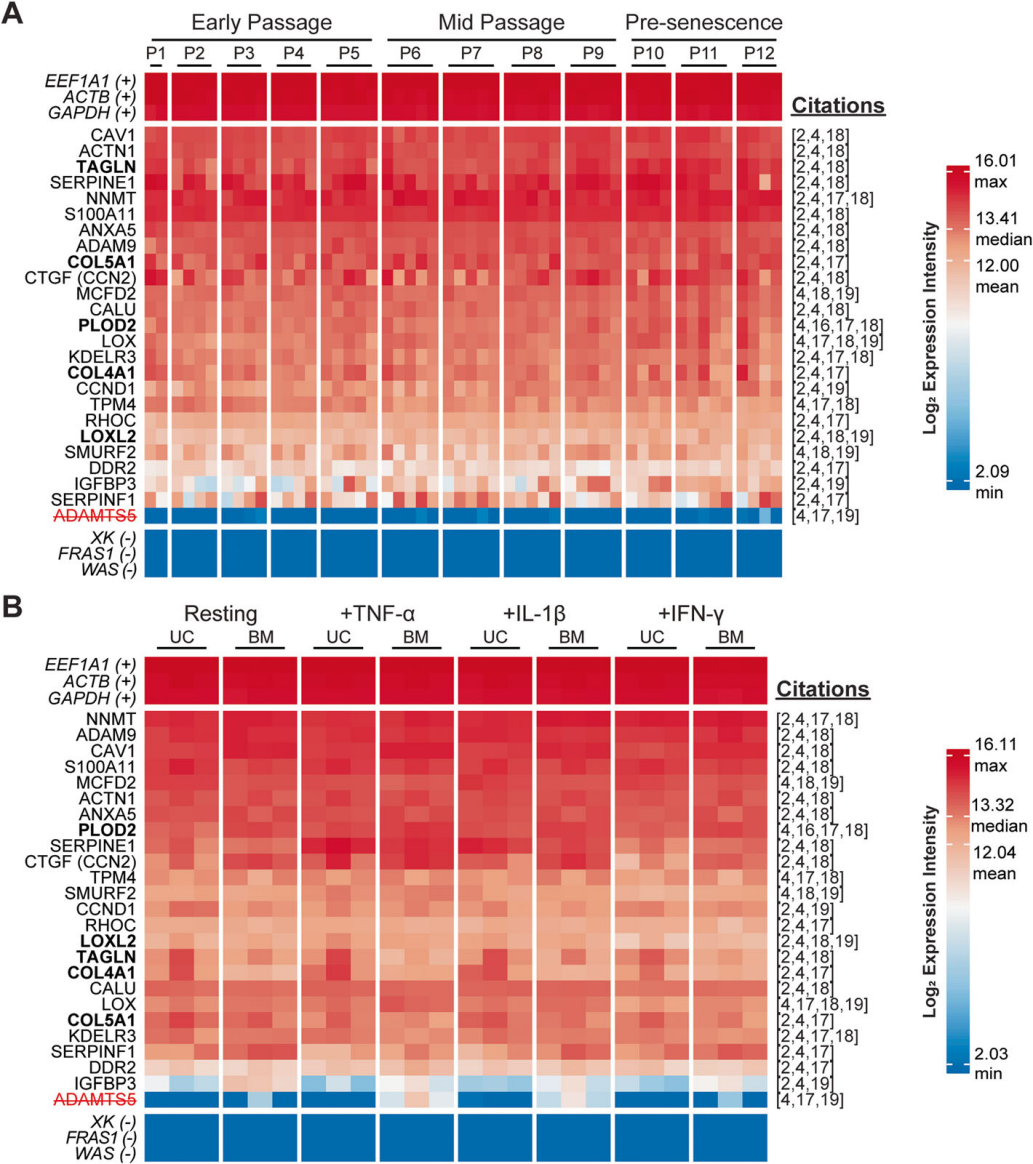
Candidate genes for a refined MSC lineage gene profile. Twenty-four genes previously determined to be specific to the MSC lineage are reliably expressed in both **a** serially expanded UC-MSCs and **b** cytokine-licensed UC and BM-MSCs. *ADAMTS5* is undetected in multiple samples in each data set and is not suitable as an enduring marker gene. Genes in bold are upregulated in MSCs versus fibroblasts [2, 16]. Italicized genes are positive (+) and negative (−) controls, the latter of which have involvement in genetic disease

Four hundred and thirty-three Roson-Burgo signature genes and 4 Rohart MSC test genes were expressed across our 2 data sets, and together represent an enduring lineage profile (Table 1, Additional file 1: Fig. S1, Additional file 2: Fig. S2). MSC gene signatures tabulated by Roson-Burgo et al. [4] reveal that 25 of their 433 genes were previously validated in 2 or more studies [2, 16–19] (Fig. 3). Of the 4 Rohart MSC test genes reliably expressed in our data, only *PRRX1* was independently cited as an MSC identity gene [17]. The panel of 25 genes was detected at moderate to high levels across our data sets, except for *ADAMTS5* (Fig. 3). Of these, *COL4A1*, *COL5A1*, *LOXL2*, *TAGLN*, and *PLOD2* are known to be upregulated in MSCs versus fibroblasts [2, 16]. Stability of this refined panel of 24 MSC lineage genes confirmed here and by others [2, 4, 16–19] supports its utility for standardized assays.

## Discussion

Recent advances in MSC therapies have revealed a need for reliable and accessible MSC identity tests to support cell production [20]. Two independent groups have proposed MSC transcriptomic signature profiles that purportedly distinguish MSCs from other stromal and stem cells [4, 5], potential tools that could address this gap. Despite deep and rigorous data mining to elucidate these signatures, no genes are common to both profiles. To our knowledge, there has been no reported use of the signature proposed by Roson-Burgo et al. [4]. Literature citing Rohart et al. [5] report successful Rohart MSC test scores for either comparative qualification of cell samples [21, 22] or for examining effects of chondrogenic differentiation [23]. Thus, transcriptomic profiling is a nascent MSC classification tool. Here, we queried whether MSC gene signatures are applicable under the pressures of routine MSC manipulation including cell expansion and immune licensing by interrogating two recent transcriptome data sets encompassing these parameters.

The human UC and BM-MSCs evaluated here are commercially available as starting material for cell therapy products and meet the International Society of Cell and Gene Therapy minimal criteria for MSCs [1]. All resting MSC populations had near-perfect compliance with the comprehensive Roson-Burgo panel, whether they were cultured in chemically defined or hPL-supplemented media. This signature gene profile proved largely stable, as few genes were affected by cell aging or licensing. Seven genes, *ADAMTS5, DACT1, IGFBP5, ISLR, LAMA4, LMO7,* and *SMIM14*, proved unreliable in both data sets, and 5 others were unreliable either during cell expansion or following activation. Reliable detection of 433 signature genes across the test matrix validates their utility as MSC identity genes. The Rohart MSC test is available for datasets submitted to Stemformatics (www.stemformatics.org) [24, 25], but current wait times for curation and analysis limit its accessibility, particularly for industrialized processes. Eleven of the 16 proposed signature genes were represented in the array data. Interestingly, only 41% of these markers were reliably detected in our test matrix. Whether these 4 Rohart MSC genes can still distinguish MSCs from similar cell types, for example using the “bootsPLS” R package also developed by Rohart et al. [26], is unknown.

Our results support transcriptomics as a potential classification tool for MSCs, using lineage genes that are stably detected through pre-senescence. Cell surface markers [1] are not ideal MSC classifiers since they can be downregulated with passaging [27], and their relative abundance can vary by MSC origin and between donors [27, 28]. Tri-lineage differentiation reportedly has substantially more impact on the proposed MSC transcriptome signatures than our cytokine licensing experiments [2, 5, 23]. In this study, we identified a core panel of MSC signature genes with minimal donor and tissue origin influence that remain stable in multiple licensing conditions.

A defined transcriptomic signature would support existing minimal criteria to improve characterization of MSC master cell banks, manufactured products, and intermediates. A refined gene profile amenable to small-scale quantitative polymerase chain reactions (qPCR) is more accessible than microarray and next-generation sequencing technologies for product screening. Although the size and proprietary test framework of the Rohart MSC test set [5] is attractive for this purpose, many genes were not represented or were unexpressed in our data set. By contrast, 91% of the Roson-Burgo signature genes were available in our test matrix, and 97% of which were reliably detected. Overall, 437 (96%) of the 456 analyzed lineage genes were expressed during culture manipulation. Curation of these findings using supporting data from previously reported MSC lineage studies reveals 24 genes with potential utility as an MSC identity test for rapid, standardized, and cost-effective MSC product characterization by qPCR. This gene panel represents a substantial refinement to commercially available MSC identity tests, one of which uses markers purportedly identified in a single study of BM-MSCs and CD34+ hematopoietic precursors [29, 30]. By contrast, the panel of 24 candidate genes validated here includes 5 markers shown to be upregulated in MSCs versus fibroblasts in multiple studies and may be particularly useful to estimate fibroblast contamination*.*

## Supplementary information

**Additional file 1.**

**Additional file 2.**

## Data Availability

The microarray data used to generate these results are available at the NCBI Gene Expression Omnibus [9, 10] through accession numbers GSE119987 and GSE129165.

## Abbreviations

BM: Bone marrow
hPL: Human platelet lysate
MSC: Mesenchymal stromal cell
P: Passage
*p*: *p* value
qPCR: Quantitative polymerase chain reaction
UC: Umbilical cord
