# Supplementary figures and images for: Transcriptome profiles acquired during cell expansion and licensing validate mesenchymal stromal cell lineage genes
Source: Stem Cell Res Ther. 2020 Aug 14;11:357. doi: 10.1186/s13287-020-01873-7 (PMC7427746; doi:10.1186/s13287-020-01873-7)

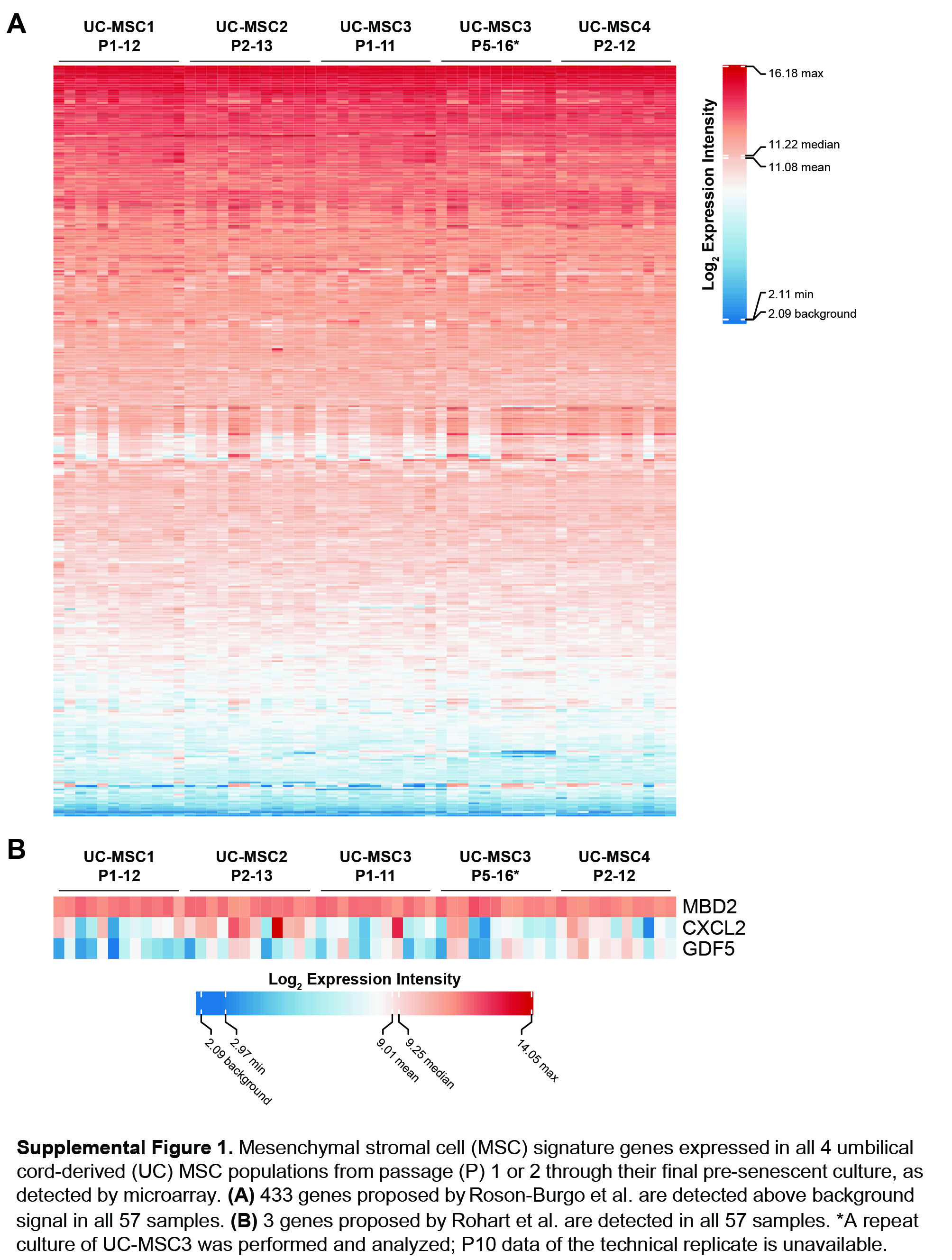

Supplement: Supplementary file 1 — Additional file 1. [file 13287_2020_1873_MOESM1_ESM.tif]

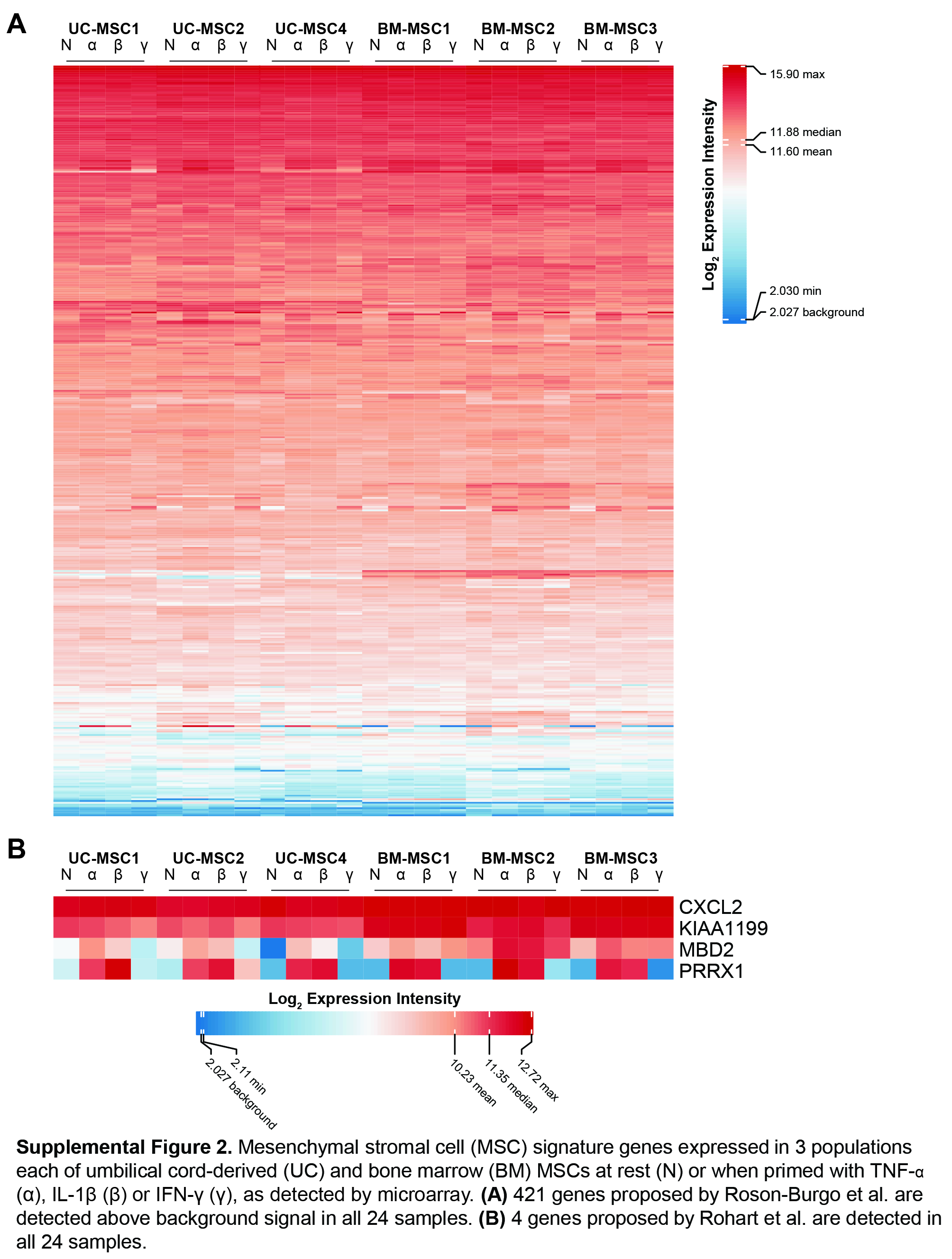

Supplement: Supplementary file 2 — Additional file 2. [file 13287_2020_1873_MOESM2_ESM.tif]
